# Learning Playing Piano with Bionic-Constrained Diffusion Policy for Anthropomorphic Hand

**DOI:** 10.34133/cbsystems.0104

**Published:** 2024-05-17

**Authors:** Yiming Yang, Zechang Wang, Dengpeng Xing, Peng Wang

**Affiliations:** ^1^ Institute of Automation, Chinese Academy of Science, Beijing, China.; ^2^ School of Artificial Intelligence, University of Chinese Academy of Sciences, Beijing, China.; ^3^ Centre for Artificial Intelligence and Robotics, Hong Kong Institute of Science and Innovation, Chinese Academy of Sciences, Hong Kong, China.

## Abstract

Anthropomorphic hand manipulation is a quintessential example of embodied intelligence in robotics, presenting a notable challenge due to its high degrees of freedom and complex inter-joint coupling. Though recent advancements in reinforcement learning (RL) have led to substantial progress in this field, existing methods often overlook the detailed structural properties of anthropomorphic hands. To address this, we propose a novel deep RL approach, Bionic-Constrained Diffusion Policy (Bio-CDP), which integrates knowledge of human hand control with a powerful diffusion policy representation. Our bionic constraint modifies the action space of anthropomorphic hand control, while the diffusion policy enhances the expressibility of the policy in high-dimensional continuous control tasks. Bio-CDP has been evaluated in the simulation environment, where it has shown superior performance and data efficiency compared to state-of-the-art RL approaches. Furthermore, our method is resilient to task complexity and robust in performance, making it a promising tool for advanced control in robotics.

## Introduction

Anthropomorphic hand manipulation is a challenging frontier in robotics due to the high degree of freedom and complex inter-joint coupling inherent [1,2]. However, it offers an excellent opportunity to exploit embodied intelligence for advanced robotic control. Recent advancements in deep learning have led to an increasing interest among researchers in exploring the potential of reinforcement learning (RL) to enhance the task complexity and variety of anthropomorphic hand manipulation [3–6], yet there remains a discrepancy in how current methods utilize the embodied intelligence of anthropomorphic hands to facilitate learning.

Existing research on RL for anthropomorphic hand manipulation has focused more on reward shaping [7,8] and prior knowledge guidance [9,10], and little attention has been paid to how the structural properties of the anthropomorphic hand can help the control algorithm. Human hand movements involve 31 different muscles, and at least 25 degrees of freedom [11], while a typical anthropomorphic hand has over 20 degrees of freedom [12]. Assuming that, in RL policy, the hand joints are conditionally independent and are modeled equally in the action space, it would needlessly complicate anthropomorphic hand manipulation. However, if we provide a more suitable action space for the complex joint control and employ policy with better capabilities, we can obtain better control policies.

The diffusion probability model addresses our requirement for such a network with powerful representational capabilities. As a class of generative models, diffusion probability models can transform a simple initial distribution into a complex target distribution through a sequence of small, random steps. Diffusion probability models have strong conditional generation capabilities, and their efficacy has been repeatedly demonstrated in areas such as image generation and denoising [13–16]. Recently, diffusion probability models have been incorporated into the RL domain as a novel policy representation method capable of handling complex and multi-modal action distributions. This distinguishes them from traditional unimodal policies and enhances the policy’s expressiveness, thereby enabling agents to make more informed decisions [17–19].

Furthermore, some bionic constraints between the joints can simplify our modeling of the anthropomorphic hand action space. For instance, an extension of the distal joint remains impossible when fingers are flexed, which means that the connections between the joints in one finger are tighter than those in the others [11]. It is logical to take this into account when modeling the action space of the anthropomorphic hand manipulation.

**Fig. 1.**
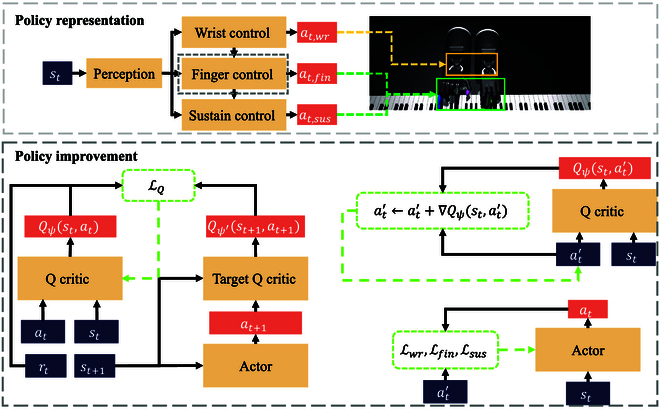
Method overview. In the policy representation part, our approach divides anthropomorphic hand joints into 3 groups based on their correlation with human hand control. For policy improvement, we innovatively propose iterative control-based action gradient to achieve joint optimization of 3 sub-action heads.

In this paper, we introduce a novel off-policy RL algorithm for anthropomorphic hand manipulation, based on the bionic control properties of the human hand and diffusion probability model. The framework of the approach is illustrated in Fig. 1. The anthropomorphic hand control is modeled as an iterative control problem. The algorithm optimizes the design of the action space and configures sub-policies with diffusion probability models of greater representation ability, making full use of the structural properties of the anthropomorphic hand. We will describe it further in the “Policy representation via bionic-constrained action” section. To improve the policy, instead of using the commonly used policy gradient in actor–critic methods, we propose an innovative iterative control-based action gradient, which we elaborate in the “Policy improvement of Bio-CDP” section. This approach enables more effective policy improvement and contributes to the overall performance of our algorithm.

Our objective is to create an RL algorithm that excels in anthropomorphic hand manipulation scenarios by considering the bionic structural properties of anthropomorphic hands. Our work makes 3 contributions toward achieving this goal:

• We propose a novel RL algorithm specialized for anthropomorphic hand manipulation, named the Bionic-Constrained Diffusion Policy (Bio-CDP), which demonstrates superior performance and data efficiency.

• To the best of our knowledge, we are the first to incorporate the bionic properties of the human hand in modeling anthropomorphic hand manipulation in RL, leading to a more natural and efficient policy representation.

• To tackle the challenges of anthropomorphic hand manipulation, we combine RL with diffusion policy. This effectively improves the learning process and results in a better performance.

## Preliminaries and Background

### Reinforcement learning

Reinforcement learning is commonly formulated as a Markov Decision Process (MDP) [20]. MDP is defined as a tuple $\left(S,A,r,P,\gamma \right)$, where ***S*** represents the state space, and A represents the action space. The reward function is denoted as r:S×A→ℝ. The transition probability function $P:S\times A\times S\to \left[0,1\right]$ defines the probability of transitioning between states. The discount factor *γ* ∈ [0, 1) is used to discount future rewards. The policy $\pi :S\times A\to \left[0,1\right]$ denotes the probability of selecting action *a* in state *s*. The objective of the agent’s policy *π* is to maximize the expected cumulative discounted reward $J\left(\pi \right)=E_{\tau ∼\pi }\left[\sum_{t=0}^{\infty }‍\gamma^{t}r\left(s_{t},a_{t}\right)\right]$, where ***τ*** = (*s*_0_, *a*_0_, *s*_1_, *a*_1_, ⋯) represents the trajectory induced by policy *π*. Additionally, in the field of RL, we commonly define the state-action value function *Q^π^* and the value function *V^π^* as follows:

$$\begin{array}{l} Q^{\pi }\left(s_{t},a_{t}\right)=E_{s_{t+1},a_{t+1},\cdots }\left[\sum_{l=0}^{\infty }‍\gamma^{l}r\left(s_{t+l}\right)\right],\\ \;V^{\pi }\left(s_{t}\right)=E_{a_{t},s_{t+1},\cdots }\left[\sum_{l=0}^{\infty }‍\gamma^{l}r\left(s_{t+l}\right)\right], \end{array}$$

where *a_t_* ∼ *π*(*a_t_*| *s_t_*), *s*_*t* + 1_ ∼ ***P***(*s*_*t* + 1_| *s_t_*, *a_t_*) for *t* ≥ 0.

When deep RL is used for robot manipulation tasks, robot movement is controlled by a continuous action space that controls each joint. Deep RL policies typically model continuous action space as the Gaussian distribution to play action [21–23]:

$$a_{t}∼N\left(\mu \left(s_{t}\right),\sigma \left(s_{t}\right)\right)$$ (1)

where *μ*(*s_t_*) and *σ*(*s_t_*) are the mean and variance of the output of the policy *π* in the state *s_t_*.

### Diffusion probability model

In the realm of deep learning, the diffusion probability model emerges as a latent variable generative model, designed to approximate an undisclosed *p*-dimensional data distribution, denoted as *p_data_* [24]. This model incorporates 2 processes—the forward process and the reverse process, collaborating to accurately approximate the target distribution. For the sake of brevity, our discussion here will be confined to the continuous-time diffusion process [25].

The forward process {*x_n_*}_*n*=0:*N*_ is governed by a stochastic differential equation

$${dx}_{n}=f\left(x_{n},n\right)dn+g\left(n\right){d\omega }_{n},$$ (2)

where *ω_n_* signifies the standard Wiener process. The terms *f*(*x_n_*, *t*) and *g*(*n*) are referred to as the drift and diffusion terms respectively in the diffusion process, thereby formulating the transition distribution $p_{n_{0}}\left(x_{n}|x_{0}\right)=N\left(x_{n}|\sqrt{\alpha_{n}}x_{0},{\left(1-\alpha_{n}\right)}^{2}I\right)$for *α_n_* ∈ [0, 1] and $p_{N}\left(x_{N}\right)=N\left(x_{N}|0,I\right)$. This implies that as the diffusion process unfolds, the random variable *x*_0_, which follows the data distribution *p_data_*(*x*), is transformed progressively into a standard Gaussian distribution $x_{N}∼N\left(0,1\right)$ through the additive noise.

In contrast, the reverse process adheres to a reverse stochastic differential equation

$$dx_{n}=\left[\;f\left(x_{n},n\right)-g^{2}\left(n\right)\nabla \text{log}p_{n}\left(x_{n}\right)\right]dn+g\left(n\right)d\omega_{n},$$ (3)

where *p_n_*(*x_n_*) denotes the marginal probability distribution of the random variable *x_n_* at step *n* ∈ [0, *N*]. It is through the parameterization and iterative learning of the score function ∇ log *p_n_*(*x_n_*) in the reverse process that we accomplish the recovery of the data distribution *x*_0_ ∼ *p_data_* from the standard Gaussian distribution $x_{N}∼N\left(0,1\right)$.

### Related Works

In this section, we will discuss two aspects: 1) the combination of the diffusion probability modeland reinforcement learning, and 2) explore the research related to reinforcement learning based onanthropomorphic hand manipulation.

### Diffusion probability model in RL

Reinforcement learning has adopted diffusion probability models as a novel way of representing policies [26]. Due to their strong generative abilities, diffusion probability models are capable of modeling complex and multi-modal distributions [24]. This makes them a great fit for serving as policies in RL, as they can capture intricate action distributions beyond simple Gaussian policies [27].

Diffuser [26] was the first to combine diffusion probability models and RL. It implemented offline RL for diffusion policies through iterative denoising trajectory planning. Subsequent research has modeled the RL policy as a return conditional diffusion probability model [28], while others have chosen to model the action distribution directly with the diffusion probability model [29–31].

The diffusion probability model’s powerful ability to characterize distributions has piqued the interest of more and more RL researchers. It has been explored and applied to varying degrees in multi-task offline RL [32,33], multi-agent offline RL [34,35], trajectory generation [36,37], and data augmentation [38,39].

While diffusion probability models have made significant contributions to offline RL, online RL presents significant challenges due to its dynamic and evolving nature. The main problem is that effectively adapting the diffusion probability model to changing data distributions requires a large amount of new data [39]. Yang et al. [17] proposed the action gradient method, which applies the diffusion probability model to online RL for the first time. This provides us with a more powerful network of policies in dexterity manipulation-based RL.

### Anthropomorphic hand manipulation with RL

Deep RL for robot anthropomorphic hand manipulation has seen a surge in interest due to remarkable advancements in deep learning. Researchers are exploring the use of RL for anthropomorphic hand manipulation. For anthropomorphic hand manipulation, haptic sensing is paramount to achieve closed-loop control. Integrating tactile perception with RL, as highlighted in recent studies [40], could offer complementary advantages, such as enhanced adaptability and improved handling of unstructured environments. The work of Andrychowicz and his team is a significant achievement in this area. They accomplished the task of controlling Rubik’s cubes by utilizing RL [6]. Following this breakthrough, many researchers have conducted further studies to enhance our understanding of the manipulation of dexterity in more depth and breadth [41].

Anthropomorphic hand manipulation is a complex task that makes it difficult to learn directly from sparse reward. To overcome this, many studies have focused on applying reward-shaping to anthropomorphic hand manipulation. Christen et al. [42] proposed a parameterized multi-objective reward function to solve this problem. Garcia-Hernando et al. [8] came up with a physics-based approach, while Ma et al. [7] proposed an automated reward-shaping method that uses large language models. All studies demonstrated how well-designed reward functions can effectively guide the learning process toward successful manipulation.

Another line of research focused on incorporating prior knowledge about the environment and task into DRL algorithms for anthropomorphic hand manipulation. For instance, Li et al. [43] proposed a Learning from Demonstration (LfD) approach that combined imitation learning with DRL to improve the performance of a robotic arm in complex manipulation tasks. Similarly, work by Ze et al. [9] and Arunachalam et al. [10] used human hand data to guide the learning process. Their findings revealed that incorporating prior knowledge can significantly enhance the performance of RL algorithms in anthropomorphic hand manipulation.

Despite these advancements, existing DRL methodologies for anthropomorphic hand manipulation have overlooked the inherent intricacies of the problem, such as biomechanical constraints and intrinsic structures of hand control. To address this gap, we propose Bio-CDP, a novel DRL algorithm designed explicitly for anthropomorphic hand manipulation that integrates biomechanical constraints and intrinsic structures into the RL policy. Our proposed methodology has been evaluated within the *RoboPianist* framework [44] and has outperformed cutting-edge RL approaches for anthropomorphic hand control.

## Methods

**Fig. 2.**
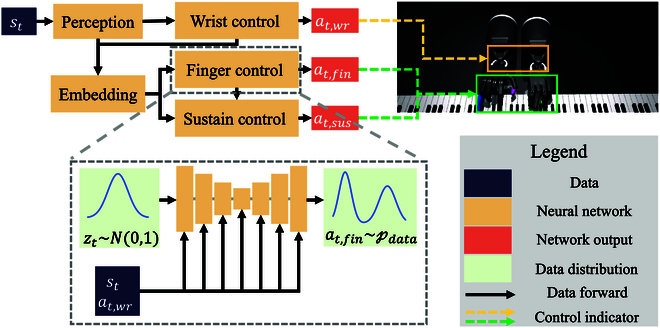
Policy representation in Bio-CDP. We decompose the action space for anthropomorphic hand control by first playing the coarse-grained workspace through wrist control, and then playing the fine-grained actions such as fingers and sustained tones based on wrist actions. The particularly complex finger control is modeled by a diffusion policy.

**Fig. 3.**
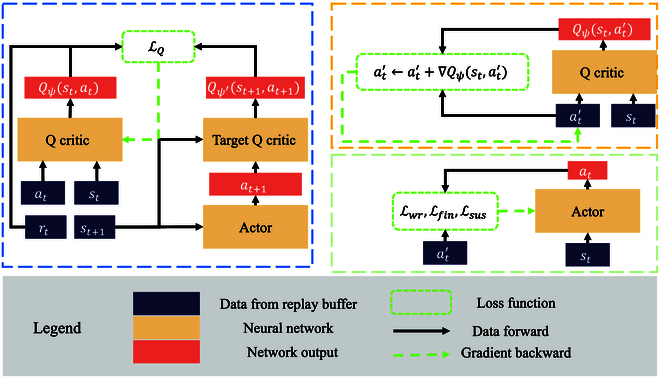
Bio-CDP policy improvement framework. The blue dashed box shows the critic network update; the orange dashed box shows the action gradient framework; and the green box shows the actor network update.

In this section, we present the details of our approach in the following 2 aspects: policy representation (as shown in Fig. 2) and policy improvement (as shown in Fig. 3).

### Policy representation via bionic-constrained action

To better model high-dimensional continuous actions, our approach uses the actor–critic framework with stochastic policy. As illustrated in Fig. 2, we have created a novel policy representation for the anthropomorphic hand that is inspired by human hand control. In human hand control, we first determine the position and posture of the hand through the arm and wrist, and then realize the precision operation by controlling the fingers. Inspired by this, we set up the anthropomorphic hand control policy by first making decisions about wrist movements to determine the hand posture, and then subsequent controls such as finger joints and sustained tones based on the wrist movements. On the one hand, based on the intrinsic structure of the action space, we decompose the originally complex action space into a number of relatively simple sub-action spaces, which reduces the difficulty of policy modeling and learning. On the other hand, this structure of iterative control can provide more valuable information to the relatively complex sub-policies so that they can take more valuable actions. The control of the anthropomorphic hand is divided into 4 components. The initial part is the perception layer, which utilizes a multilayer perceptron (MLP) to receive and process current environmental and proprioceptive state *s_t_*. The resulting processed information is then utilized by all subsequent action heads.

The wrist joint of the human hand primarily controls the general space of hand manipulation, while the fingers are responsible for fine manipulation tasks [45,46]. Therefore, the hand joint space A is divided into 3 action heads: control of the wrist joint $A_{\mathrm{wr}}$, control of the finger joints $A_{\mathrm{fin}}$, and task-related sustaining control $A_{\mathrm{sus}}$. The decision is made by the wrist control sub-policy *π_wr_*(·| *s_t_*) to determine the workspace of the anthropomorphic hand by making a wrist action *a*_*t*,*wr*_ based on the current state. From there, the finger control sub-policy *π_fin_*(·| *s_t_*, *a*_*t*,*wr*_) handles the finger aspect of the fine operation *a*_*t*,*fin*_. Lastly, the sustain sub-policy *π_sus_*(·| *s_t_*, *a*_*t*,*wr*_, *a*_*t*,*fin*_) outputs the action *a*_*t*,*sus*_ that decides whether or not to delay the sound. For wrist and sustain control with small action space, we select MLP as the parametric sub-policy using Eq. 1.

By incorporating bionic constraints informed by the anatomy and motion dynamics of the human hand, our method refines the action space to align with the natural affordances and constraints of anthropomorphic hands. This integration not only streamlines the policy search but also ensures that the resultant control strategies are inherently synergistic with the physical form and function of the robotic hand.

Considering the control complexity and the direct impact on task completion, we propose utilizing the diffusion policy as a sub-policy for finger motion control. Diffusion policy generates actions through a stochastic process, distinguishing it from value-based and parametric policy representation.

Similar to Eq. 2, we use a sequence {(*a_n_*| *s*)}_*n*=0:*N*_ to model the forward process of the diffusion sub-policy *π_fin_*(·|*s*) for any state *s*. Here, *a*_0_ denotes the finger action distribution, while *a_N_* denotes the standard Gaussian distribution. The process is defined as follows:

$$a_{n,\mathrm{fin}}|a_{0}∼N\left(e^{-n}a_{0},\left(1-e^{-2n}\right)I\right).$$ (4)

Obviously, when *n* is large enough, the forward process transforms the policy *π_fin_*(·|*s*) to a normal Gaussian distribution $z∼N\left(0,1\right)$.

As shown by the gray box in Fig. 2, in policy inference, the diffusion policy uses the reverse process. This means that we have to recover the finger action distribution from the standard Gaussian distribution based on the additional condition (current state *s_t_*). To do this, we assume a step size of *h* > 0, a reverse length of $K=\frac{N}{h}$, and set *k* = *hk*, *k* = 0, 1, …, *K*. This allows us to divide the interval [0, *N*] into K equal parts. Using this approach, we can obtain a recursive formula for the reverse diffusion process in discrete form [47], given by:

$$a_{k+1}=e^{h}a_{k}+\left(e^{h}-1\right)\left(a_{k}+2\hat{S}\left(a_{k},s,N-k\right)\right)+\sqrt{e^{2h}-1}z$$ (5)

Here, $\hat{S}(\cdot ,s,N-k)$ represents an approximate of the score function at a given state *s*. We can define it as follows:

$$\hat{S}\left(\cdot ,s,N-k\right)=\text{arg}\underset{\hat{s}\in F}{\text{min}}\;E_{a∼\pi_{k}(\cdot |s)}\left[{\left\|\hat{s}(a,s,k)-\nabla \log\left(\pi_{k}(a|s)\right)\right\|}_{2}^{2}\right]$$ (6)

Here, F is the collection of function approximations. In our approach, it is the finger control head in Fig. 2.

Researchers have demonstrated the convergence of the diffusion probability model as an RL policy, and they have shown that the diffusion policy has a strong ability to characterize multimodal distributions [17–19]. Compared to an unimodal policy, a multimodal policy enhances the expressiveness of complicated policy, allowing agents to make more informed decisions.

After generating finger actions *a_fin_*, we enforce consistency in the contraction and relaxation of the joints on each finger by constraining the actions. To achieve this, we clip the corresponding finger action to 0, which is the joints on a finger that is diastolic when the basal joint on that finger is contracted. Similarly, we do the opposite when the basal joint is relaxed.

### Policy improvement of bio-CDP

In order to improve policy, we have employed the action gradient approach [17], as the diffusion probability model can only fit policy distribution. This approach differs from traditional online RL algorithms in that it not only fits the policy distribution but also improves it. Our policy improvement process can be seen in Fig. 3, which comprises 3 main stages: critic network update, action improvement, and actor network update. These stages are represented by blue boxes, orange boxes, and green boxes, respectively. Each will be described in detail below.

#### Critic update

During the first stage of the training process, we focus on learning the Q network, also known as the critic network. To achieve this, we use the clipped double-Q network, as proposed in [48]. The objective function for updating the network parameters is to minimize the squared difference between the predicted *Q* value and the target *Q* value. The target *Q* value is computed as the sum of the immediate reward received in the current state and the discounted minimum *Q* value of the next state, calculated using either one of the 2 independent sub-networks *ψ*_1_ and *ψ*_2_. This design helps to mitigate the problem of over-estimation of *Q* values.

$${L\;}_{Q}={\left\|Q_{\psi_{i}}\left(s_{t},a_{t}\right)-\left(\;r\left(s_{t+1}|s_{t},a_{t}\right)+\gamma \underset{\;i=1,2}{\text{min}}\;Q_{\psi_{i}^{\prime }}\left(s_{t+1},a_{t+1}\right)\;\right)\right\|}_{2}^{2}.$$ (7)

The Q network has a set of parameters *ψ*, which is updated during training. In addition, we have a target Q network with parameters $\psi^{\prime }$ , which is updated using the Exponential Moving Average approach. The update equation for the target Q network is given as:

$$\psi \prime =\tau \cdot \psi +\left(1-\tau \right)\cdot \psi \prime .$$ (8)

Here, *τ* ∈ [0, 1] controls the target Q network update step size. This approach ensures that the parameters of the target network do not change too much during training, thereby improving the stability of learning.

#### Action improvement

When using the action gradient for RL, the policy updates based on *Q* estimation are not directly applied to the actor network. Instead, the actions stored in the replay buffer are updated using gradient ascent in the direction of ∇*_a_Q^π^*(*s*, *a*)|_*a* = *π*(*s*)_. This update is performed using the following equation:

$${\left.a_{t}^{\prime }\leftarrow a_{t}^{\prime }+\eta \nabla_{a}Q_{\psi }\left(s_{t},a\right)\right|}_{a=a_{t}^{\prime }},$$ (9)

where *η* is the learning rate.

The action improvement process is performed iteratively, with each iteration updating the actions in the replay buffer based on the current Q-function estimates. We will store an additional set of improvement actions $a_{t}^{\prime }$ corresponding to the current state *s_t_* in the replay buffer. These improvement actions are then used in the next stage of the training process and actor network update, where the actor network is trained to produce actions similar to these improved ones. This iterative process of action improvement and actor update eventually leads to the development of a policy that can generate actions leading to higher expected returns.

#### Actor update

After implementing action improvement in the “Action improvement” section, the action network uses supervised learning to fit state-action pairs. This means that the wrist control head and the sustain control head update the network by minimizing the mean square error (MSE) through the following equations:

$$\begin{array}{ll} & L_{\mathrm{wr}}={\left\|{\hat{a}}_{t,\mathrm{wr}}-a_{t,\mathrm{wr}}^{\prime }\right\|}_{2}^{2},\\ & \;L_{\mathrm{sus}}={\left\|{\hat{a}}_{t,\mathrm{sus}}-a_{t,\mathrm{sus}}^{\prime }\right\|}_{2}^{2}. \end{array}$$ (10)

Here, sub-policies $\pi_{\mathrm{wr}}^{\theta }\left(s_{t}\right)$ and $\pi_{\mathrm{sus}}^{\theta }\left(s_{t}\right)$ output actions ${\hat{a}}_{t,\mathrm{wr}}$ and ${\hat{a}}_{t,\mathrm{sus}}$, respectively, parameterized by *θ*.

As for the finger control head, we use a U-Net similar to Denoising Diffusion Probabilistic Models (DDPM) [24]. In the diffusion policy, the actions outputted by the network are obtained by continuously performing a reverse diffusion process over an initial Gaussian noise *z*; therefore, the optimization objective of the diffusion probability model is not the MSE concerning the distribution of the target actions, but rather the fitting of the portion of the denoising process that is subtracted from the random noise. According to the derivation of Yang et al. [17], we can obtain the optimization objective of the finger control head as follows

$$L_{\mathrm{fin}}={\left\|z-\epsilon_{\theta }\left(\sqrt{{\bar{\alpha }}_{k}}a_{t}^{\prime }+\sqrt{1-{\bar{\alpha }}_{k}}z,s,k\right)\right\|}_{2}^{2}.$$ (11)

Here $\sqrt{{\bar{\alpha }}_{k}}$ and $\sqrt{1-{\bar{\alpha }}_{k}}$ are signal rate and noise rate, respectively, where ${\bar{\alpha }}_{k}\in \left(0,1\right)$ is the coefficient that varies with the diffusion step, on which we can define the noise schedule. We use the cosine schedule defined by Nichol and Dhariwal [49]. The *ϵ_θ_* is the learnable part in the finger control sub-policy network, which means that we change from the conventional prediction of the mean to the prediction of the noise.

To summarize the information presented above, we can provide our Algorithm 1.


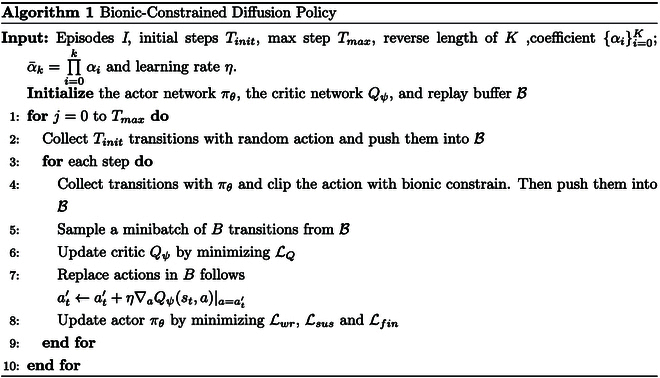


## Experiments

We conducted experiments to evaluate and analyze Bio-CDP. In the “Experimental setting” section, we provide a detailed description of the experimental setting. In the “Performance advantages of Bio-CDP” section, we demonstrate the superiority of our approach by comparing Bio-CDP with methods that are widely used in RL, as well as state-of-the-art methods. In the “Ablation study” section, we perform an ablation study on Bio-CDP.

### Experimental setting

We conduct experimental validation based on the *RoboPianist* [44], an RL virtual environment for controlling anthropomorphic hands to play music on a piano. The skill of piano playing is also relatively difficult for the human hand, requiring complex finger coordination and planning skills and high temporal and spatial precision, and some difficult songs even challenge the limits of human limits of dexterity. Therefore, choosing *RoboPianist* as a validation environment can be a good way to test the performance of our method on high-dimensional anthropomorphic hand manipulation.

**Fig. 4.**
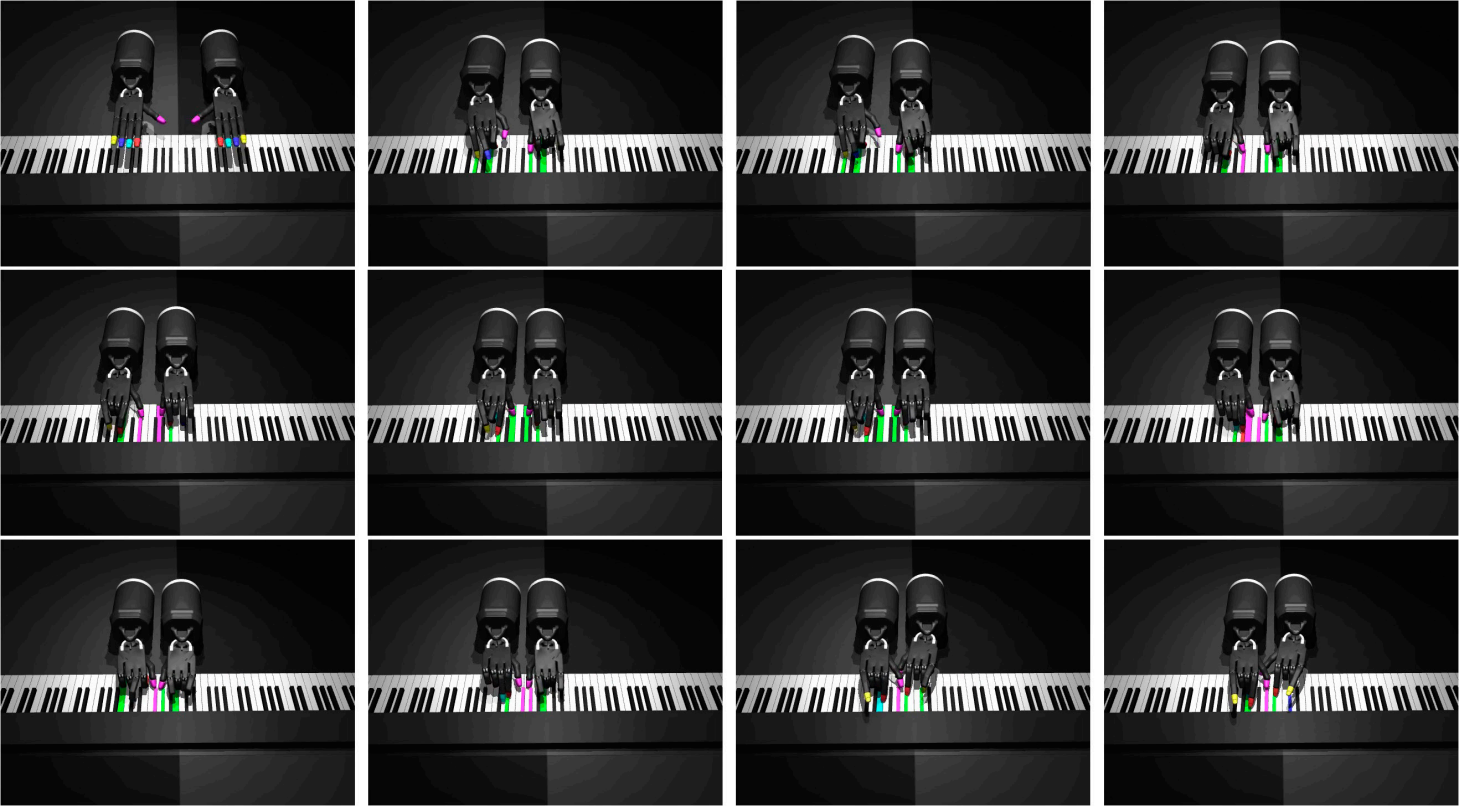
Snapshots of the anthropomorphic hand playing piano.

In Fig. 4, we can see a snapshot of the *RoboPianist* environment. The agent has a state observation dimension of 1,126, and the lookahead horizon is set to 10. The action space consists of 45 dimensions, which includes the left and right hand for each of the 22 joints, as well as a sustained voice control. The finger control head employs an MLP with 4 (2 for other control heads) hidden layers and non-linear activation functions to approximate the diffusion process. The specific configuration includes 4 hidden layers with 256 units each, using Mish [50] for activation. The critic network is a 4-layer MLP with 256 hidden units, using Mish as the non-linear activation function. The RL training hyperparameters specific to our method are shown in Table. Other hyperparameters and environment settings we keep consistent with the default settings in the environment.

**Table.** Hyperparameters for Bio-CDP

Name	Value	Description
Batch size	256	Batch size for each training episode
*γ*	0.8	Discount factor for future rewards
*τ*	0.05	Coefficient in Eq. 8
*N*	100	Diffusion steps
Agent learning rate	5 × 10^−4^	Learning rate for actor and critic
*η*	3 × 10^−2^	Learning rate for action gradient in Eq. 9
Noise ratio	1.0	Noise ratio in sample process

### Performance advantages of bio-CDP

To evaluate the effectiveness of our method, we conducted a comparison of Bio-CDP with 3 tasks in *RoboPianist* featuring different pieces:

• *C Major Scale*: This is the simplest piece, as it only involves playing the white keys, with no black keys involved. It is usually the first scale beginners learn to play on the piano.

• Chopin’s *Nocturne in E-flat major, Op. 9, No. 2*: This piece is of intermediate difficulty, requiring some technical skills and performance experience to play smoothly. Its melody and chord transitions are much more complex than the C Major Scale. For robot agents, the main challenge is primarily in dynamic control due to its rapid playing tempo and extensive dynamic range.

• Debussy’s *Clair de Lune*: This piece is relatively more complex technically and musically, making it the most challenging of the three. It poses challenges chiefly in polyphonic playing and sustaining notes. This piece demands more nuanced coordination between multiple fingers and precise control over the sustain, making it relatively more challenging within our anthropomorphic hand piano-playing framework.

The difficulty of playing these 3 pieces of music increases in turn. In these environments, we compared the following 4 methods:

Bio-CDP: Our proposed method in Algorithm 1.

DroQ: Comparison method, the current state-of-the-art offline RL algorithm [51].

Soft Actor-Critic (SAC): Comparison method, the most widely used offline RL algorithm currently [22].

Proximal Policy Optimization (PPO): Comparison method, the most widely used online RL algorithm currently [23].

**Fig. 5.**
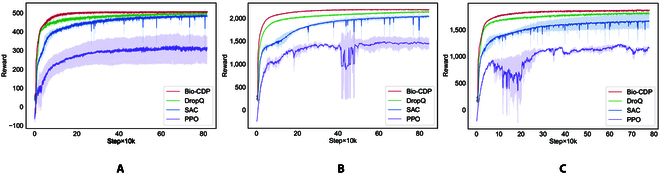
Training curves for each method. The results are averaged from 3 random seeds. The horizontal axis denotes the number of steps trained and the vertical axis denotes the cumulative reward for a round. (A) Playing *C Major Scale*. (B) Playing *Nocturne in E-flat major, Op. 9, No. 2*. (C) Playing *Clair de Lune*.

The results of our experiments are shown in Fig. 5. The red curve in the figure is our proposed Bio-CDP, the green curve is DroQ, the blue curve is SAC, and the purple curve is PPO. It can be seen that the learning efficiency and the final performance of our method are the best in all 3 scenarios. As the tune becomes harder, the other baseline methods more or less degrade in convergence speed and algorithmic stability, while our method is basically unaffected, converging to near-optimal performance at around 100k steps.

### Ablation study

**Fig. 6.**
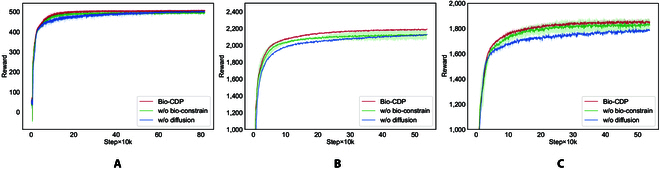
Ablation study for Bio-CDP. The results are averaged from 3 random seeds. The horizontal axis denotes the number of steps trained and the vertical axis denotes the cumulative reward for a round. (A) Playing *C Major Scale*. (B) Playing *Nocturne in E-flat major, Op. 9, No. 2*. (C) Playing *Clair de Lune*.

Results from the ablation study, illustrated in Fig. 6, underscore the significance of each module in the Bio-CDP method when applied to *Clair de Lune* and *Nocturne in E-flat major, Op. 9, No. 2*. The full method, represented by the red trajectory in Fig. 6, is compared against 2 variants: one without the biological constraints (“w/o bio-constrain”, green line) and one replacing the diffusion probability component with an MLP (“w/o diffusion”, blue line).

When conducting ablation experiments, we observed that the performance differentiation in simple environments (*C Major Scale*) was not significant enough. This phenomenon is most likely due to the low learning threshold of the simple task so that even after removing certain model components, the remaining models are still able to learn enough policies to complete the task. In other words, simple environments do not require many aspects of the model, so the effect of the ablation component is not significant.

However, in more complex environments such as Chopin’s *Nocturne in E-flat major, Op. 9, No. 2* and Debussy’s *Clair de Lune*, the strengths of our approach become apparent. Our Bio-CDP shows its superior performance in processing these musical works with higher degrees of freedom and more complex inter-finger coordination requirements. Compared to the baseline RL approach, Bio-CDP can learn effective control policies faster on these complex tasks, and the learned policies are more accurate and robust in execution. This result emphasizes the effectiveness of our approach in dealing with high-dimensional continuous control tasks and confirms our original design intent of integrating human hand control knowledge with extended policy expression capabilities.

## Conclusion and Future Work

In this paper, we have presented a novel approach, Bio-CDP, for RL in anthropomorphic hand manipulation tasks. By integrating bionic constraints and diffusion policy, our method effectively captures the structural properties of anthropomorphic hands and enhances the expressibility of the policy. Our experimental results in the simulation environments have demonstrated the effectiveness and superiority of Bio-CDP in terms of performance and data efficiency. Moreover, our approach remains robust and resilient even as the complexity of tasks increases.

However, while Bio-CDP shows promising results, there are still several areas for further exploration and improvement. (a) Although the simulation results for Bio-CDP are promising, there are still many challenges waiting to be addressed when transitioning from simulation to reality. (b) The design of more refined bionic constraints that model the human hand’s control properties more accurately could potentially improve the performance of our method. (c) Exploring the application of diffusion policy in other high-dimensional continuous control tasks could broaden the scope of our method beyond anthropomorphic hand manipulation. These directions will be our future focus as we continue to advance the field of RL for anthropomorphic hand manipulation.

## Data Availability

The code used for this study is openly available in https://github.com/sachiel321/Bio-CDP. All data needed to evaluate the conclusions in the paper are present in the paper and/or the Supplementary Materials. Additional data related to this paper may be requested from the authors.
